# Apoptosis inhibitor of macrophage suppress immune responses via IL-10 production and delay bacterial clearance in *Mycobacterium avium* infection

**DOI:** 10.3389/fcimb.2025.1578082

**Published:** 2025-05-05

**Authors:** Chiaki Kajiwara, Ayako Shiozawa, Satoko Arai, Tetsuo Yamaguchi, Sohei Harada, Toru Miyazaki, Kazuhiro Tateda

**Affiliations:** ^1^ Department of Microbiology and Infectious Diseases, Faculty of Medicine, Toho University School of Medicine, Tokyo, Japan; ^2^ Marsico Lung Institute, School of Medicine, University of North Carolina at Chapel Hill, Chapel Hill, NC, United States; ^3^ The Institute for AIM Medicine, Tokyo, Japan

**Keywords:** apoptosis inhibitor of macrophage, CD5L, foamy macrophage, *Mycobacterium avium*, lipid metabolism, interleukin 10

## Abstract

Non-tuberculous mycobacteria infections, including *Mycobacterium avium*, are increasingly recognized as a growing public health concern, even among immunocompetent individuals. These infections are a significant cause of chronic pulmonary disease, and they are characterized by the formation of foamy macrophages (FMs) that facilitate bacterial persistence. Previously, we reported that apoptosis inhibitor of macrophage (AIM), a protein secreted by macrophages, promotes lipid droplet accumulation in *M. avium*-infected macrophages. However, the precise role of AIM in modulating immune responses remains unclear. This study aimed to elucidate the effect of AIM on FM formation, bacterial burden, and immune response in *M. avium*-infected mice by comparing AIM knockout (KO) mice with wild-type mice. Histological analysis revealed a reduction in FM formation in AIM KO mice, accompanied by decreased lipid droplet accumulation and altered expression of lipid metabolism-related genes. Furthermore, AIM KO mice exhibited a reduced bacterial load in the lungs, highlighting decreased cytokine production, including IL-1β, compared to wild-type mice. In addition, the analysis of the immune cells of AIM KO mice using flow cytometry revealed an increase in M1 macrophages and IFN-γ-producing T cells, as well as a decrease in M2 macrophages and interleukin 10 (IL-10)-producing T cells. The reduced expression of CD36 and PD-L1 in macrophages from AIM KO mice further supports the skewing toward an M1 phenotype. *In vitro* experiments with bone marrow-derived macrophages (BMDMs) confirmed reduced bacterial growth and lipid droplet formation in AIM KO BMDMs, which was restored by AIM and IL-10 treatment. These findings suggest that AIM contributes to the promotion of FM formation by establishing an immunosuppressive environment that promotes the establishment of *M. avium* through IL-10 production.

## 1 Introduction

Non-tuberculous mycobacteria are a diverse group of mycobacteria distinct from *Mycobacterium tuberculosis* (*M. tuberculosis*) complex and *M. leprae*, with over 200 species identified in soil, water, dust, and other environmental sources; they are an emerging opportunistic pathogen (Shamaei and Mirsaeidi, 2021). *M. abscessus* and *M. avium* complex (MAC), which comprises *M. avium* and *M. intracellulare*, are among the most significant causes of chronic pulmonary disease in humans (Andréjak et al., 2010; Lai et al., 2010; Moore et al., 2010; Donohue and Wymer, 2016; Lee et al., 2019). MAC infection is of public health importance, especially in Japan, where the number of patients is increasing, in contrast to the decline in the number of patients with tuberculosis (Namkoong et al., 2016). Despite the increasing prevalence of MAC infection, the underlying pathophysiology is poorly understood.

Foamy macrophages (FMs) are specialized immune cells that accumulate lipid droplets and play an important role in the pathogenesis of mycobacterial infections (Daniel et al., 2011; Saka and Valdivia, 2012; Johansen et al., 2019; Sachdeva et al., 2020; Agarwal et al., 2021). The stimulation of lipid-sensing nuclear receptors, Toll-like receptors, and other membrane-bound macrophage receptors by *M. tuberculosis* cell wall lipids (including oxygenated mycolic acid) is involved in the biosynthesis of FMs (Peyron et al., 2008; Bowdish et al., 2009; Dkhar et al., 2014). In C3HeB/FeJ mice, which develop granulomas with caseous necrosis upon infection with *M. tuberculosis*, numerous bacterial cells are observed in the necrotic area and in FMs (Seto et al., 2022). *M. tuberculosis* in caseum is non-proliferative (Muñoz-Elías et al., 2005; Sarathy et al., 2018), whereas *M. tuberculosis* in FMs is highly proliferative (Walter et al., 2021). Caloric restriction reduces bacterial growth and lung injury and protects mice from *M. tuberculosis* infection. These effects were associated with increased autophagy in immune cells, increased fatty acid oxidation, and decreased mTOR activity (Palma et al., 2021). Recently, in a mouse model of *M. avium* infection, mice fed a protein-restricted diet showed increased fatty acid levels and related gene expression in lung tissue, as well as increased numbers of FMs and bacteria in the lungs (Choi et al., 2024). These data suggest that changes in macrophage energy metabolism are closely related to the induction of FMs after a mycobacterial infection. Understanding the mechanisms by which FMs support mycobacterial persistence is critical for the development of novel therapeutic strategies for diseases such as tuberculosis and MAC infections.

Apoptosis inhibitor of macrophage (AIM), also known as CD5L, is a protein secreted by macrophages (Miyazaki et al., 1999). AIM plays a role in the initiation and progress of certain contemporary diseases (Kim et al., 2008; Gray et al., 2009; Yu et al., 2009; Maehara et al., 2014; Yamazaki et al., 2014; Aran et al., 2018; Okanoue et al., 2022; Sanchez-Moral et al., 2023; Takimoto-Sato et al., 2023). In addition, AIM is involved in the polarization of alternatively activated (M2) macrophages through an autophagic mechanism and in an ID3 transcription factor-dependent manner (Sanjurjo et al., 2018). Furthermore, AIM contributes to IL-10-induced anti-inflammatory responses by inhibiting the activation of the inflammasomes (Kim et al., 2021).

Macrophages infected with *M. avium* produce substantial amounts of AIM, contributing to FM formation and an increased bacterial burden in the lungs (Kajiwara et al., 2023). In this study, we aimed to evaluate the differences in responsiveness to *M. avium* infection using AIM knockout (KO) mice compared to wild-type mice and investigate the underlying lipid metabolism in macrophages.

## 2 Materials and methods

### 2.1 Laboratory animals

BALB/c mice were purchased from the Jackson laboratory Japan, Inc. (Kanagawa, Japan). AIM KO mice on BALB/c background used in this study were provided by Dr. Miyazaki of the Institute for AIM Medicine. Experiments were performed on 8- to 12-week-old mice. All mice were housed under specific-pathogen-free conditions in the animal care facility of Toho University School of Medicine (Tokyo, Japan). All experiments were performed according to the guidelines for Proper Conduct of Animal Experiments (Science Council of Japan). The animal and pathogen protocols were approved by the Institutional Care and Use Committee (No. 23-531, No. 23-149).

### 2.2 *M. avium* inoculation and determination of bacterial counts

Clinical isolate of *M. avium* subsp. *hominissuis* strains stocked at Toho University Hospital were used for the pneumonia model. Colonies were grown on Middlebrook 7H10 (Difco, Franklin Lakes, NJ, USA) agar plates supplemented with 10% oleic acid/albumin/dextrose/catalase (OADC; Difco) at 37°C for 10–14 days. Afterward, they were suspended in saline to a McFarland Standard 1.0, which was further diluted and used. Thereafter, mice were anesthetized intramuscularly with a mixture of medetomidine hydrochloride (0.3 mg/kg), midazolam (4 mg/kg), and butorphanol tartrate (5 mg/kg). Subsequently, they were infected with approximately 1 × 10^6^ CFU/mouse intranasally. To determine bacterial counts, the right lungs of the sacrificed mice were harvested and homogenized using a homogenizer (IKA Japan K.K., Osaka, Japan) in 1 mL of saline. After a 1:10 serial dilution in saline, 10-μL aliquots of the homogenates were inoculated onto Middlebrook 7H10 agar plates supplemented with 10% OADC and incubated at 37°C for 10–14 days. The bacterial colonies were counted visually.

### 2.3 Histopathological analysis of mouse lungs infected with *M. avium*

The left lungs of the mice were removed at the indicated time points and fixed in buffered 10% formalin solution. After being embedded in paraffin wax, 6-μm-thick tissue sections were cut perpendicular to the anterior-posterior axis. The sections were placed on polylysine-treated slides and stained with hematoxylin and eosin (H&E). To detect foam cells, lungs were frozen in optimal cutting temperature (OCT) compound (Sakura Finetek, Tokyo, Japan) and 4-μm-thick tissue sections were prepared in a cryostat. The sections were fixed in 10% formalin at room temperature for 15 min. Thereafter, they were washed in phosphate buffered saline, stained with Lipi-Green according to the manufacturer’s protocol (Dojindo, Kumamoto, Japan), and finally sealed with a DAPI-containing mounting medium (Vector Laboratories, Burlingame, CA, USA). The fluorescence intensities of intracellular lipids were analyzed using a Carl Zeiss LSM 710 confocal microscope and quantified using ImageJ software (National Institutes of Health, Bethesda, MD, USA).

### 2.4 Fite staining for paraffin-embedded mouse lung tissue sections

Paraffin sections were deparaffinized using oil xylene (peanut oil:xylene = 1:2), followed by rehydration through graded ethanol to distilled water. Sections were stained using carbol fuchsin solution at room temperature for 30 min without heating. After washing with distilled water, decolorization was performed using 1% HCl in ethanol. Subsequently, the sections were rinsed with water and counterstained with methylene blue. Finally, slides were washed, dehydrated, cleared, and mounted for microscopic examination to observe acid-fast bacteria.

### 2.5 Bone marrow-derived macrophage preparation and *in vitro* infection

Bone marrow-derived macrophages (BMDMs) were prepared as described previously (Kajiwara et al., 2018). Cells were harvested using Trypsin-EDTA (Life Technologies) and seeded in 96-well (1 × 10^5^ cells per well) or 12-well (1 × 10^6^ cells per well) flat-bottom tissue culture plates (BD Falcon) overnight. BMDMs were incubated with *M. avium* diluted in plain RPMI at a multiplicity of infection of 10 and incubated for 1.5 h. At the end of the infection period, non-phagocytosed and nonadherent bacteria were removed by washing three times with a fresh medium. At the indicated time points, culture supernatants were collected for enzyme-linked immunosorbent assay (ELISA). The infected macrophages were lysed with 0.1 mL of distilled water to count the viable bacterial number, as previously described (Kajiwara et al., 2018). In some experiments, recombinant AIM (R&D Systems, Minneapolis, MN, USA) at concentrations of 10–1000 ng/mL or IL-10 (BioLegend, San Diego, CA, USA) at 20 ng/mL was added. These were applied 1 h prior to infection and at the start of culture after infection.

### 2.6 Isolation of lung cells and flow cytometric analysis

Cells were isolated from lung tissue as previously described (Kajiwara et al., 2020). To detect live cells, the cells were stained with Zombie Violet dye (BioLegend). The washed cells were mixed with staining buffer (PBS containing 2% fetal bovine serum and 2 mM EDTA) and incubated on ice for 15 min with Fc receptor blocker (anti-mouse CD16/32, clone 93) to block non-specific reactions. Subsequently, the cells were washed with staining buffer and surface-stained for 30 min on ice using experimentally designed combinations of the following antibodies: PerCP/Cy5.5 anti-mouse CD11b (clone M1/70), FITC anti-mouse Ly6G (clone 1A8), PE anti-mouse Ly6G (clone 1A8), PE/Cy7 anti-mouse F4/80 (clone BM8), APC anti-mouse PD-L1(clone 10F.9G2), AlexaFluor488 anti-mouse CD36 (clone HM36), PE anti-mouse CD86 (clone GL-1), PerCP/Cy5.5 anti-mouse CD206 (clone C068C2), FITC anti-mouse CD3e (clone 145-2C11), APC/Cy7 anti-mouse CD4 (clone RM4-5), and APC anti-mouse CD8a (clone 53-6.7). All antibodies were purchased from BioLegend. The cells were washed and fixed with 4% paraformaldehyde. Intracellular lipid droplets were stained using a Lipid Droplet Assay Kit - Blue. For intracellular cytokine staining, cells were stimulated with Cell Activation Cocktail (BioLegend) for 4 h at 37°C. Thereafter, cells were fixed and permeabilized with Cyto-Fast Fix/Perm Buffer Set according to the manufacturer’s protocol (BioLegend). After washing, cells were stained with PE anti-mouse interferon-gamma [IFN-γ] (clone XMG 1.2), PerCP/Cy5.5 anti-mouse IL-10 (clone JES5-16E3), or IgG isotype control. Subsequently, cells were analyzed using a Fortessa flow cytometer (BD Biosciences, Franklin Lakes, NJ, USA) and FlowJo software (Tree Star Inc., Ashland, OR, USA).

### 2.7 ELISA

Cytokines in mouse lung tissue or culture supernatant were measured using SP-D, IL-1β, TNF-α, IFN-γ, and IL-10 mouse ELISA kits (R&D Systems), according to the manufacturer’s protocols.

### 2.8 RNA isolation and gene expression analysis

Total RNA was isolated from mouse lungs and BMDMs using TRIzol reagent (Invitrogen, Waltham, MA, USA), according to the manufacturer’s instructions. For quantitative PCR analysis, 1 μg of total RNA was reverse-transcribed using a High-Capacity cDNA Reverse Transcription Kit (Applied Biosystems, Foster City, CA, USA). The SYBR Green RT-PCR technique was used, and the analyses were performed using QuantStudio 5 Real-Time PCR Instrument (Applied Biosystems). We used the following PCR primers: *Cd5l*, 5′-TTTGTTGGATCGTGTTTTTCAGA-3′ (forward) and 5′-CTTCACAGCGGTGGGCA-3′ (reverse); *Ascl6*, 5′- ATGCAGAGGAACTCAACTACTG-3′ (forward) and 5′- CTTTGTCCTGTCCTCGTCTTT-3′ (reverse); *Dgat1*, 5′- CCAACCATCTGATCTGGCTTAT-3′ (forward) and 5′- GACTCAGCATTCCACCAATCT-3′ (reverse); *Acat1*, 5′- GAAAAGGCCAATCTGGAACA-3′ (forward) and 5′- AGTCTCCTTTCTCCCCCAAA-3′ (reverse); *Abca1*, 5′- CCCAGAGCAAAAAGCGACTC-3′ (forward) and 5′- GGTCATCATCACTTTGGTCCTTG-3′ (reverse); *Abcg1*, 5′- CAAGACCCTTTTGAAAGGGATCTC-3′ (forward) and 5′- GCCAGAATATTCATGAGTGTGGAC-3′ (reverse); *Inos*, 5′-CCCTTCCGAAGTTTCTGGCAGCAGC-3′ (forward) and 5′-GGCTGTCAGAGCCTCGTGGCTTTGG-3′ (reverse); *Arg1*, 5′-GAACTGGCAAAAGGATGGTGA-3′ (forward) and 5′-TGTGGGTTGTTGACCTCAAAC-3′ (reverse); *Ym1*, 5′- GGGCATACCTTTATCCTGAG-3′ (forward) and 5′- CCACTGAAGTCATCCATGTC-3′ (reverse); *Il-10*, 5′- TTTGAATTCCCTGGGTGAGAA-3′ (forward) and 5′- GCTCCACTGCCTTGCTCTTATT-3′ (reverse); *Ifn-γ*, 5′-GAACTGGCAAAAGGATGGTGA-3′ (forward) and 5′-TGTGGGTTGTTGACCTCAAAC-3′ (reverse); *β-actin*, 5′ AGAGGGAAATCGTGCGTGAC-3′ (forward) and 5′ CAATAGTGATGACCTGGCCGT-3′ (reverse). The relative fold changes in transcript levels were calculated using the 2^-ΔΔ^ *^C^* ^T^ method (where CT is the threshold cycle, 40). The housekeeping gene *β-actin* served as the reference standard for the loading amount and cDNA quality.

### 2.9 Statistical analysis

All results were expressed as means ± SD. Student’s *t*-tests were used for comparisons between the two groups. One-way analysis of variance followed by Tukey’s multiple comparison tests were used for between-group comparisons. Statistical analyses were performed using the GraphPad Prism 10 software (GraphPad, La Jolla, CA, USA). Differences were considered significant at *p* < 0.05.

## 3 Results

### 3.1 FMs decreased in the lungs of AIM KO mice infected with *M. avium*

**Figure 1:**
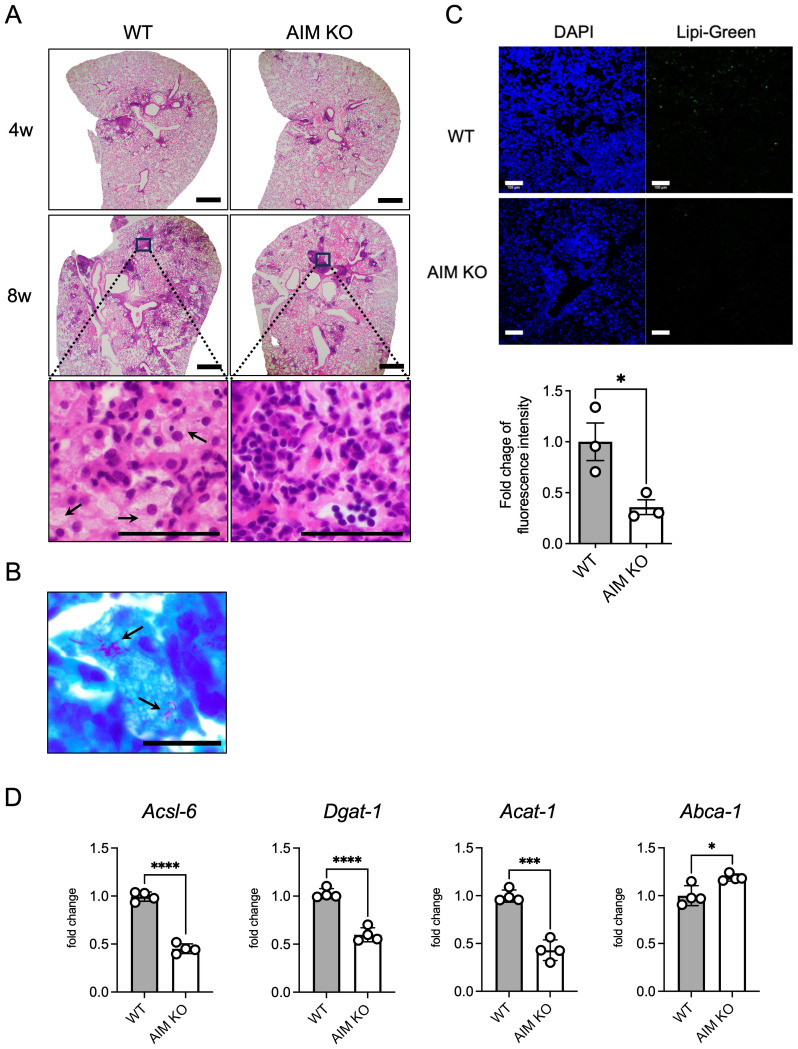
Histopathological characteristics and accumulation of foam cells in the lungs of mice infected with *M. avium*. **(A)** Representative images of H&E staining of mouse lungs 4 and 8 weeks after intranasal inoculation with 1×10^6^ CFU of *M. avium*. Scale bars: 500 μm. Arrows indicate FMs. **(B)** Bacterial localization in wild-type mouse lungs 8 weeks after infection was assessed using Ziehl-Neelsen staining. Scale bars: 20 μm. Arrows indicate intracellular mycobacteria. **(C)** Representative images of Lipi-Green staining of mouse lungs 8 weeks after intranasal inoculation with 1×10^6^ CFU *M. avium*. Scale bars: 100 μm. For quantification of Lipi-Green positive cells in the lungs, positive areas were evaluated with ImageJ Fiji software. Assessments were made in at least six randomly selected distinct regions for each mouse. Bars: means ± SEM (n = 3/group). **(D)** mRNA levels of *Acsl6*, *Dgat1*, *Acat1*, and *Abca1* in the lungs at 8 weeks after infection. Data are expressed as fold increase. Bars: means ± SD (n = 4/group). Results were confirmed using two independent experiments. *p < 0.05, ***p < 0.001, ****p < 0.0001.

To investigate whether AIM is involved in the induction of FMs in the lungs of *M. avium*-infected mice, H&E staining was performed on lung sections from AIM knockout mice (**Figure 1A**). In wild-type and AIM KO mice, lymphoid follicles were observed in the peribronchial region 4 weeks after infection. At 8 weeks after infection, wild-type mice exhibited numerous granulomas comprising FMs and lymphocytes; in contrast, AIM KO mice displayed developed lymphoid follicles (**Figure 1A**). The detection of *M. avium* via acid-fast bacillus staining in the lungs of wild-type mice 8 weeks after infection showed that most of the bacteria were localized in FMs (**Figure 1B**). In the detection of intracellular lipids using fluorescent markers, reduced lipid droplet accumulation was observed in AIM KO mice compared to wild-type mice (**Figure 1C**). In addition, we examined the mRNA expression levels of lipid metabolism-related factors involved in intracellular lipid droplet formation. In AIM KO mice, the expression levels of enzymes associated with lipid droplet synthesis and storage, including long-chain acyl-CoA synthetase (*Acsl6*), diacylglycerol acyltransferase 1 (*Dgat1*), and acetyl-coenzyme A acetyltransferase 1 (*Acat1*), were decreased, whereas the expression of ATP-binding cassette protein A1 (*Abca1*), involved in lipid efflux, was increased (**Figure 1D**). These results suggest that AIM contributes to the formation of foam cells following *M. avium* infection, thereby corroborating previous findings that AIM levels increased in the serum and lung tissue of infected wild-type mice and that the number of foam cells also increased (Kajiwara et al., 2023).

### 3.2 Bacterial levels decreased in the lungs of AIM KO mice infected with *M. avium*

**Figure 2:**
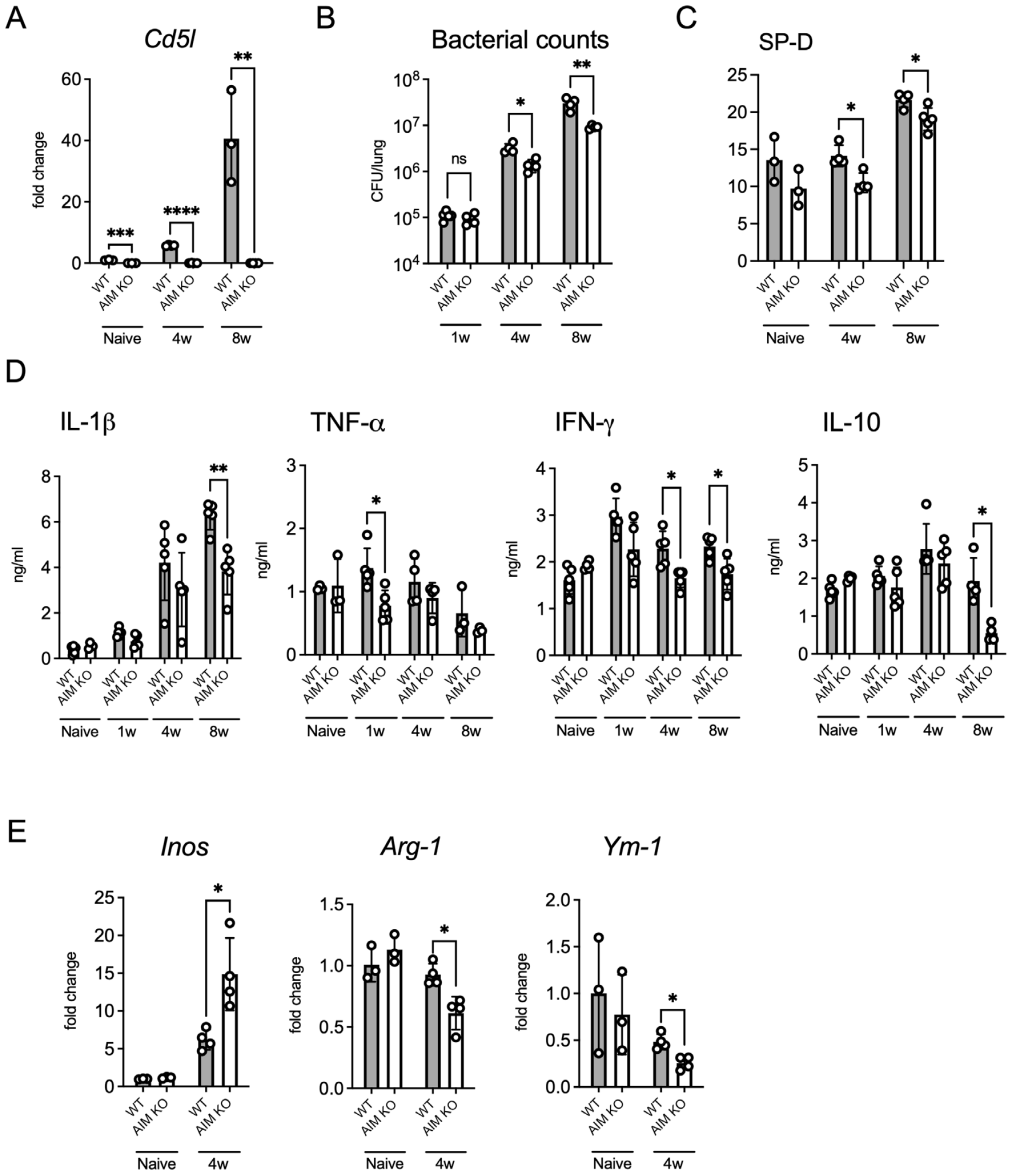
Bacterial load and cytokine profile in the lungs of mice infected with *M. avium*. **(A)** Mice were infected intratracheally with 1 × 10^6^ CFU of *M. avium*. mRNA levels of *Cd5l* in the lungs at 4 and 8 weeks after infection. Bars, mean ± SD (n = 3/group). Results were confirmed using two independent experiments. **(B)** The bacterial count in the right lungs was measured at 1, 4, and 8 weeks after infection. Bars, mean ± SD (n = 4–5/group). Results were confirmed using three independent experiments. **(C)** SP-D protein levels in the lungs at 8 weeks after infection. Bars, mean ± SD (n = 3–5/group). Results were confirmed using two independent experiments. **(D)** The protein levels of several inflammatory cytokines in the lungs were measured at 1, 4, and 8 weeks after infection. Bars, mean ± SD (n = 4–5/group). Results were confirmed using two independent experiments. **(E)** mRNA levels of *iNOS*, *Arg1*, and *Ym1* in the lungs at 4 weeks after infection. Bars, mean ± SD (n = 3–4/group). Results were confirmed using two independent experiments. *p < 0.05, **p < 0.01, ***p < 0.001, ****p < 0.0001.

We confirmed that *Cd5l* (AIM-encoding gene) mRNA expression in the lungs of WT mice increased over time following infection, which is consistent with our previous findings (**Figure 2A**) (Kajiwara et al., 2023). To investigate the effect of AIM on bacterial growth in the lungs of mice, we examined the bacterial count in the lungs of wild-type and AIM KO mice. Although no significant differences were observed between the mouse groups during the first week after infection, the bacterial load in the lungs of AIM KO mice was lower than that of wild-type mice at the fourth and eighth weeks (**Figure 2B**). The difference in bacterial load in the lungs was further highlighted by the levels of SP-D, a marker of lung damage (**Figure 2C**). Additionally, the production levels of the inflammatory cytokines IL-1β and IFN-γ, as well as the anti-inflammatory cytokine IL-10, varied depending on the proliferative characteristics of the bacteria (**Figure 2D**). Furthermore, the mRNA expression of the M1 marker *iNOS* (associated with pro-inflammatory responses) was significantly increased in AIM KO mice, whereas the mRNA expression of the M2 markers *Arg1* (related to tissue repair) and *Ym1* (associated with anti-inflammatory functions) was significantly decreased in AIM KO mice (**Figure 2E**). These findings suggest that AIM KO mice exhibit reduced lung damage and bacterial burden in response to *M. avium* infection, possibly due to enhanced M1 macrophage polarization.

### 3.3 Activated macrophages and IFN-γ-producing T cells increased in the lungs of AIM KO mice infected with *M. avium*

**Figure 3:**
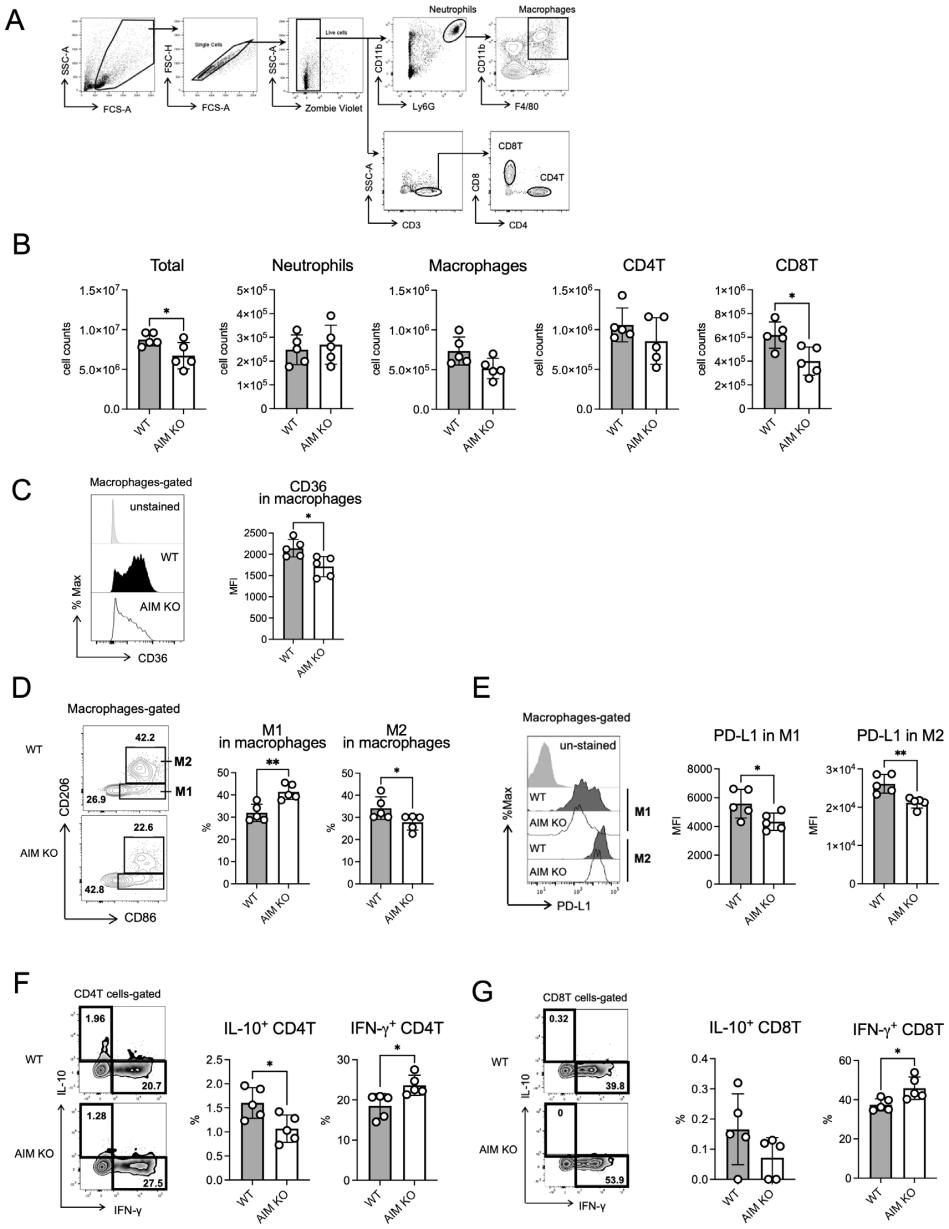
Flow cytometric analysis of lung cells from mice infected with *M. avium*. **(A)** Gating strategy for flow cytometric analysis. Cells were collected from the left lungs of mice 8 weeks after infection. The leukocyte area was gated, and the single cell population was defined as follows: neutrophils (CD11b^high^ Ly6G^high^), macrophages (neutrophils ungated, CD11b^+^ F4/80^+^), CD4T cells (CD3^+^ CD4^+^), and CD8T cells (CD3^+^ CD8^+^). **(B)** Based on the total lung cell count, the absolute numbers of neutrophils, macrophages, CD4T cells, and CD8T cells were calculated from the data of the assay shown in **(A)**. **(C)** Mean fluorescence intensity (MFI) and percentage of CD36 in macrophages in the lungs of mice 8 weeks after infection. **(D)** The CD86^+^ CD206^-^ population was defined as M1 macrophages, and the CD86^+^ CD206^+^ population was defined as M2 macrophages. **(E)** MFI of PD-L1 in M1 and M2 macrophages in the lungs of mice 8 weeks after infection. **(F)** The percentage of IL-10 or IFN-γ in CD4 T cells in the lungs of mice 8 weeks after infection. **(G)** The percentage of IL-10 or IFN-γ in CD8 T cells in the lungs of mice 8 weeks after infection. Bars, mean ± SD (n = 5/group). Results were confirmed using two independent experiments. *p < 0.05, **p < 0.01.

To investigate the differences between the immune cells in the lungs of wild-type and AIM KO mice, flow cytometry analysis was performed. Cells were isolated from the lungs of mice at 8 weeks after infection and distinguished based on cell surface markers reported previously (Kajiwara et al., 2023) (**Figure 3A**). No differences were observed in the total cell count or the number of neutrophils, macrophages, or CD4 T cells between AIM KO and wild-type mice. However, the number of CD8 T cells was lower in AIM KO mice than in wild-type mice (**Figure 3B**). Based on previously reported markers (Kakoschky et al., 2018), macrophage phenotypes were determined. Compared to wild-type mice, AIM KO mice exhibited a higher proportion of M1 macrophages, which exhibit pro-inflammatory activity, and a lower proportion of M2 macrophages, which are involved in tissue repair and anti-inflammatory responses (**Figure 3D**). Notably, the expression levels of PD-L1, a molecule that suppresses T cell activity, were reduced in both M1 and M2 macrophages in AIM KO mice (**Figure 3E**). In addition, the cell surface expression levels of CD36, which plays a role in macrophage foam cell formation (Miyazaki et al., 2011; Choi et al., 2024), was decreased in macrophages from AIM KO mice compared to wild-type mice (**Figure 3C**). Additionally, the proportion of CD4 T cells producing the pro-inflammatory cytokine IFN-γ was significantly higher in AIM KO mice than in wild-type mice, whereas the proportion of CD4 T cells producing the anti-inflammatory cytokine IL-10 was reduced (**Figure 3F**). Although the proportion of IL-10-producing CD8 T cells was very low in both groups, the proportion of IFN-γ-producing CD8 T cells was increased in AIM KO mice (**Figure 3G**). These findings suggest that in AIM KO mice infected with *M. avium*, lung macrophages are polarized toward an M1 phenotype, which enhances bacterial clearance. Furthermore, their antimicrobial activity is likely amplified by T cell-derived IFN-γ.

### 3.4 Differential expression of CD86 and PD-L1 in *M. avium*-Infected AIM KO and wild-type BMDMs

**Figure 4:**
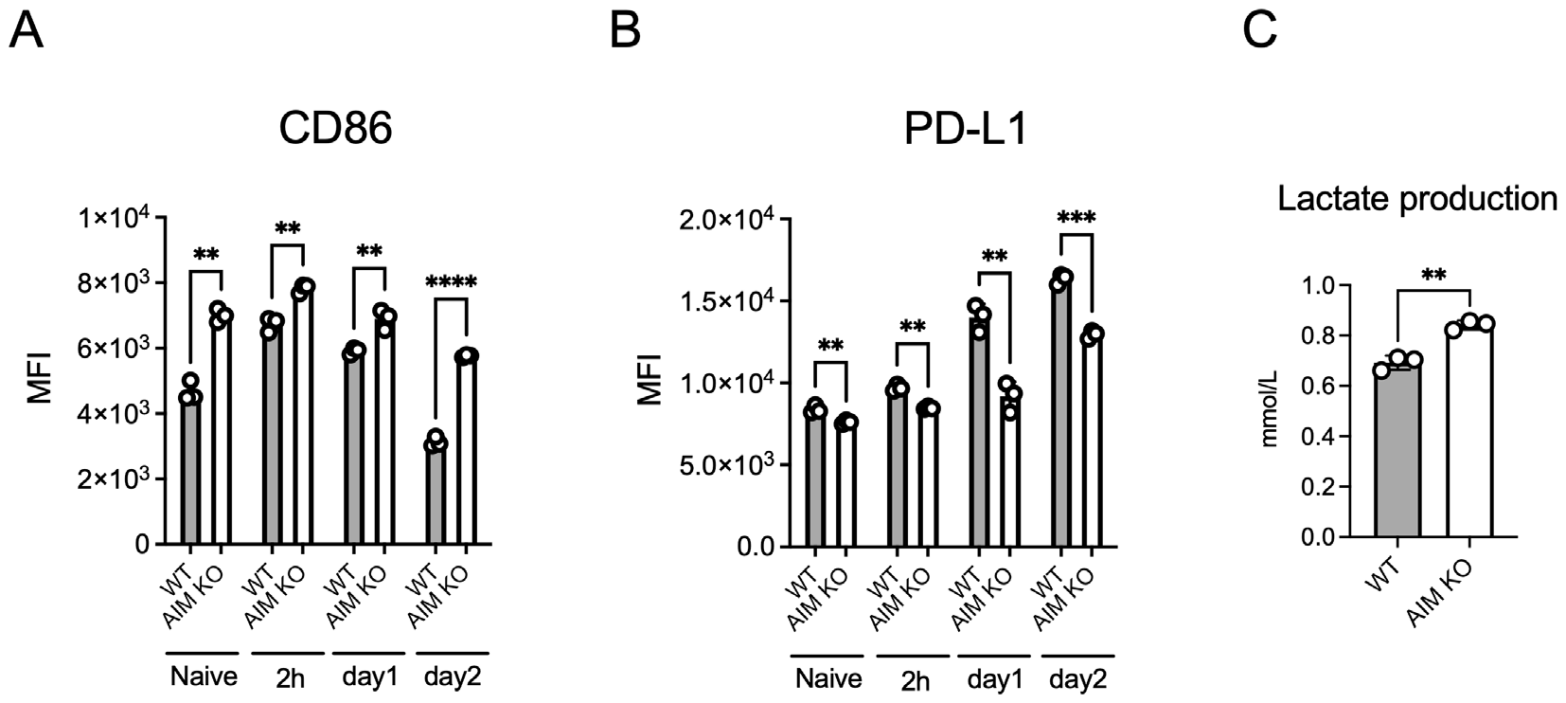
Analysis of CD86 and PD-L1 expression in BMDMs infected with *M. avium*. BMDMs infected with *M. avium* at an MOI of 10 for 1.5 h were washed and incubated. Cells were stained with anti-CD86 **(A)** or anti-PD-L1 **(B)** antibodies at the indicated time points and analyzed using flow cytometry. **(C)** The amount of lactate in the culture supernatant collected from BMDMs 2 days after infection was measured. Bars, mean ± SD, in triplicates. Results were confirmed using three independent experiments. **p < 0.01, ***p < 0.001, ****p < 0.0001.

Based on the previous *in vivo* experiment, which showed a higher proportion of M1 macrophages in the lung macrophages of infected AIM KO mice compared to wild-type mice, we investigated whether this finding could be replicated in BMDMs. The expression level of CD86, a marker of M1 macrophages, remained consistently higher in AIM KO BMDMs than in wild-type BMDMs, including in the uninfected state and up to 2 days after infection, with the difference increasing over time (**Figure 4A**). Additionally, in both BMDM types, CD86 expression was higher at 2 h and 1 day after infection than in the naive state; however, on the second day, the expression level decreased below that of naive BMDMs. In contrast, CD206, a marker of M2 macrophages, was not detected at any time point (data not shown). The expression level of PD-L1 was consistently lower in AIM KO BMDMs compared to wild-type BMDMs, in contrast to the pattern observed with CD86 (**Figure 4B**). To assess whether glycolysis was enhanced in AIM KO BMDMs, we measured the extracellular secretion of lactate, the end product of glycolysis, since M1 macrophages predominantly rely on glycolysis for energy production (Freemerman et al., 2014). The lactate concentration in the culture supernatant was significantly higher in AIM KO BMDMs (**Figure 4C**). These findings suggest that AIM promotes the M1 macrophage phenotype in *M. avium*-infected BMDMs.

### 3.5 Intracellular lipid droplets were reduced in *M. avium*-Infected AIM KO BMDMs

**Figure 5:**
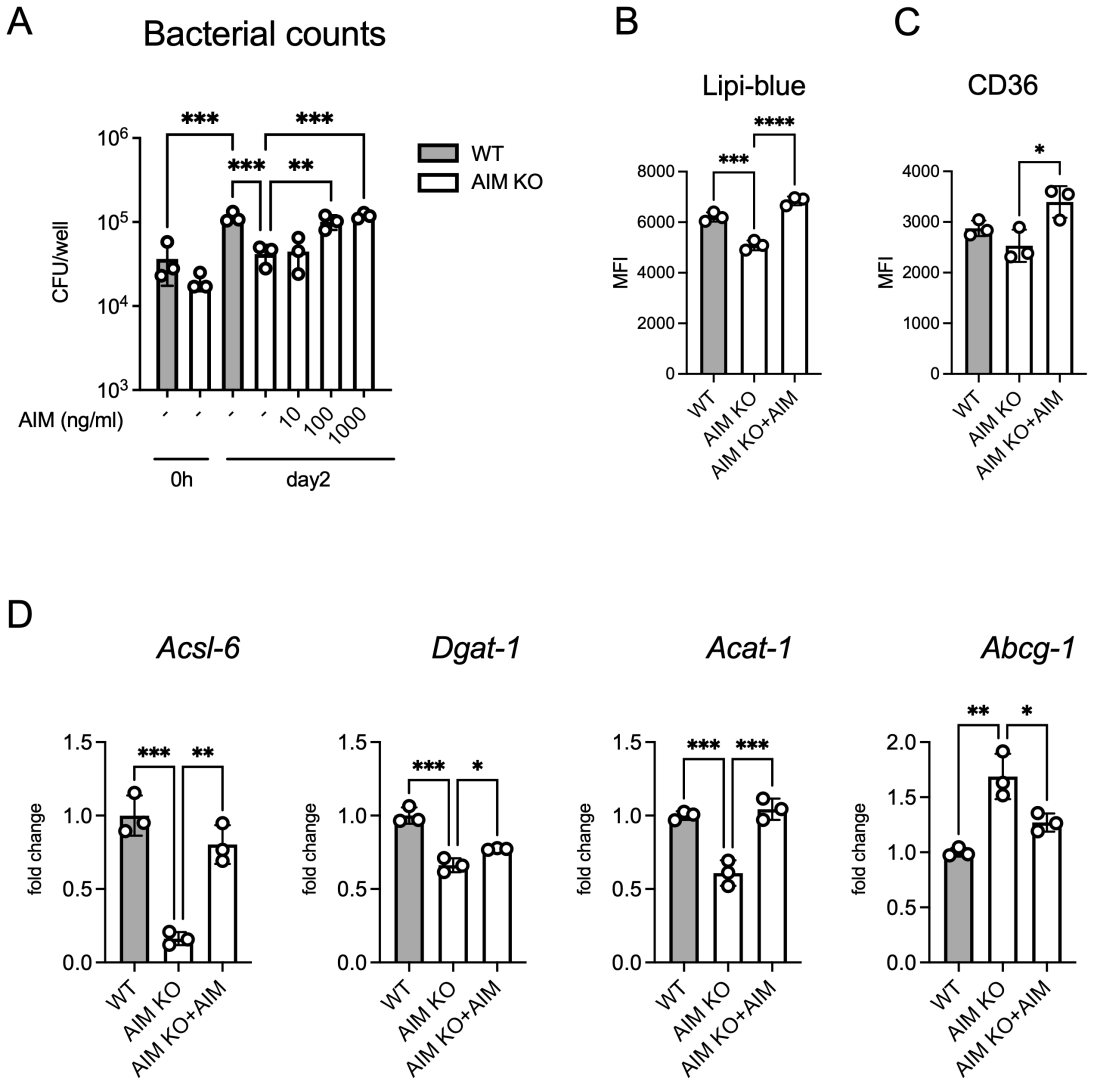
Analysis of bacterial counts and lipid accumulation in BMDMs infected with *M. avium*. **(A)** The number of bacteria in BMDMs treated with AIM at the indicated concentrations, with bacterial load evaluated 2 days after infection. **(B)** MFI of Lipi-Blue in BMDMs treated with AIM (200 ng/mL), with analysis performed 2 days after infection. **(C)** MFI of CD36 in BMDMs treated with AIM (200 ng/mL), with analysis performed 2 days after infection. **(D)** mRNA levels of *Acsl6*, *Dgat1*, *Acat1*, and *Abcg1* in BMDMs 2 days after infection, following treatment with AIM (200 ng/mL). Data are expressed as fold increase. Bars, mean ± SD, in triplicates. Results were confirmed using three independent experiments. *p < 0.05, **p < 0.01, ***p < 0.001, ****p < 0.0001.

We evaluated intracellular bacterial growth. AIM KO BMDMs exhibited significantly lower bacterial growth compared to wild-type macrophages, and the addition of recombinant AIM increased the intracellular bacterial burden in a concentration-dependent manner (**Figure 5A**). Following infection, AIM KO BMDMs demonstrated reduced intracellular lipid droplet formation and lower cell surface expression of CD36 compared to wild-type BMDMs (**Figures 5B, C**). The mRNA levels of genes associated with lipid droplet formation exhibited similar changes to those observed in the *in vivo* experiments (**Figure 5D**). These findings corroborate with the *in vivo* data, suggesting that AIM contributes to the formation of lipid droplets in macrophages.

### 3.6 IL-10 increased intracellular lipid droplets in *M. avium*-Infected AIM KO BMDMs

**Figure 6:**
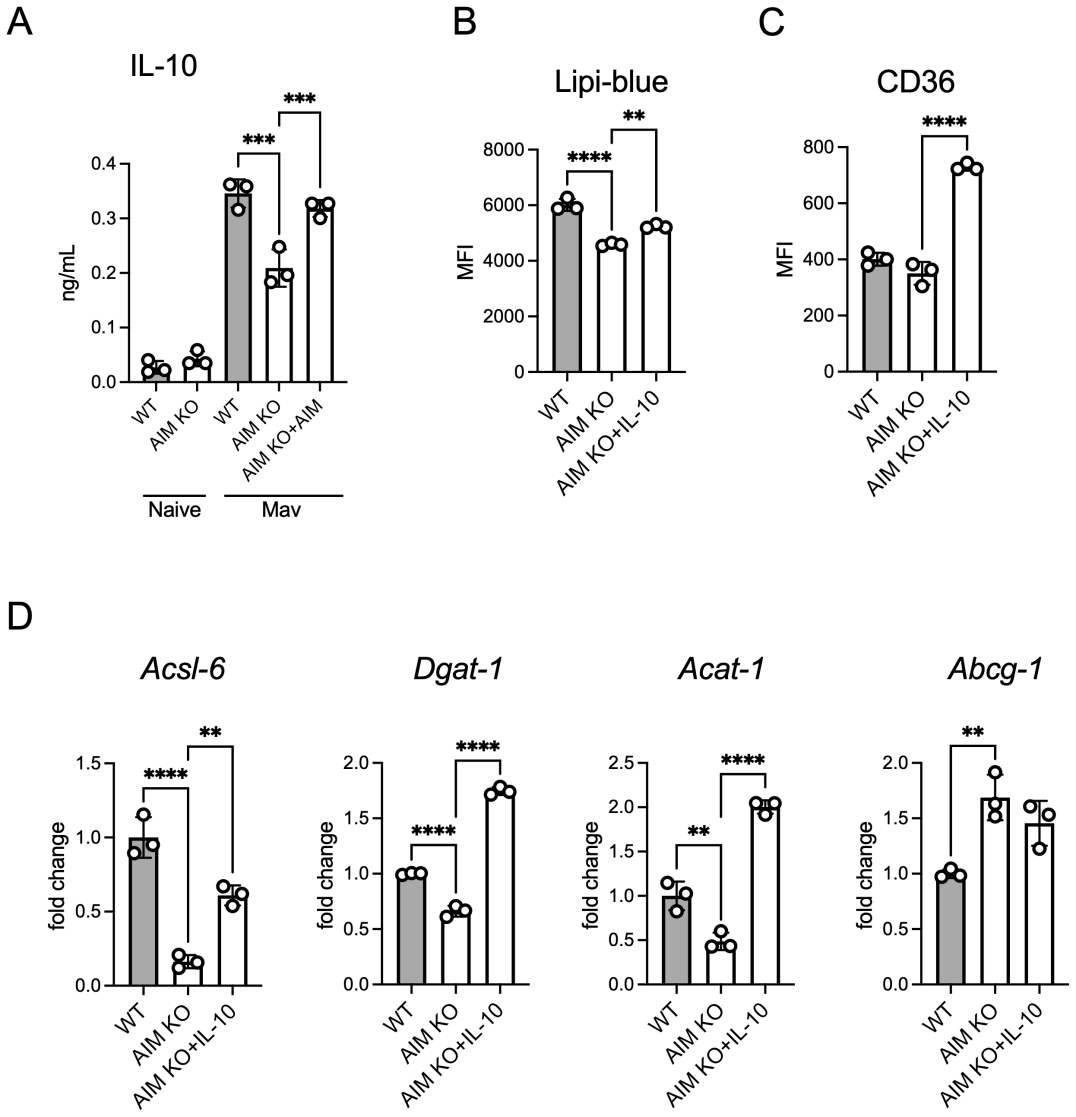
Effect of AIM on IL-10 production and lipid accumulation in BMDMs infected with *M. avium*. **(A)** Culture supernatants were collected from BMDMs, and IL-10 levels were measured using ELISA 2 days after infection, following treatment with AIM (200 ng/mL). **(B)** MFI of Lipi-Blue in BMDMs treated with IL-10 (20 ng/mL), with analysis performed 2 days after infection. **(C)** MFI of CD36 in BMDMs treated with IL-10 (20 ng/mL), with analysis performed 2 days after infection. **(D)** mRNA levels of *Acsl6*, *Dgat1*, *Acat1*, and *Abcg1* in BMDMs 2 days after infection, following treatment with IL-10 (20 ng/mL). Data are expressed as fold increase. Bars, mean ± SD, in triplicates. Results were confirmed using three independent experiments. **p < 0.01, ***p < 0.001, ****p < 0.0001.

IL-10 promotes lipid droplet formation in *M. tuberculosis*-infected human macrophages (Genoula et al., 2018). To investigate this finding in the context of *M. avium*, we assessed IL-10 levels in the culture supernatants following an infection. AIM KO BMDMs exhibited lower IL-10 secretion compared to WT BMDMs, and the addition of AIM restored IL-10 levels (**Figure 6A**). Furthermore, the addition of AIMs to AIM KO BMDMs increased intracellular lipid droplet accumulation (**Figure 6B**) and surface expression of the lipid uptake receptor CD36 (**Figure 6C**). To assess whether IL-10 affects the expression of lipid metabolism-related factors (**Figure 4E**), we examined their expression levels. IL-10 treatment resulted in an upregulation of factors involved in lipid droplet synthesis, with the exception of *Abcg1* (**Figure 6D**). These findings suggest that IL-10 promotes lipid droplet formation in *M. avium*-infected macrophages by enhancing lipid uptake and the expression of important factors involved in lipid droplet synthesis.

## 4 Discussion

This study elucidates the critical role of AIM in the pathogenesis of *M. avium* infection, particularly in relation to FMs formation and lipid metabolism, in part through the induction of IL-10 production. Our findings demonstrate that AIM is an important regulator of macrophage polarization and lipid droplet accumulation, which in turn affects bacterial burden and immune response in the lungs.

In the context of murine models of bacterial infection, a body of research has emerged regarding the potential effect of AIM on the progression of pathological conditions, with both positive and negative findings. In particular, in models of *Staphylococcus aureus* infection and sepsis, AIM has been implicated in the induction of excessive immune responses, which may contribute to the exacerbation of systemic inflammation and disease progression (Gao et al., 2019b, a). Conversely, in models of fungal peritonitis, the inflammatory response following zymosan administration is significantly increased in the absence of AIM, suggesting that AIM deficiency may cause persistent inflammation in the peritoneum owing to impaired clearance of necrotic debris (Tomita et al., 2017). This phenomenon can be attributed, at least in part, to the function of AIM as a scavenger protein, which can form complexes with various pathogens and cellular debris, thereby promoting phagocytosis (Martinez et al., 2014; Arai et al., 2016). The interaction between AIM and *M. avium* remains unclear; however, if AIM facilitates bacterial uptake by macrophages, it may provide a habitat for the bacteria, considering the ability of mycobacteria to impair the host’s bactericidal capacity (Awuh and Flo, 2017).

Flow cytometry analysis revealed that AIM KO mice had increased proportion of IFN-γ-producing CD4 and CD8 T cells and decreased IL-10 production. This cytokine profile suggests that AIM may regulate immune responses by promoting an anti-inflammatory environment, potentially facilitating bacterial survival. Interestingly, IFN-γ levels in lung tissue were reduced in AIM KO mice at 4 weeks after infection, despite more efficient bacterial clearance. This may reflect reduced antigenic stimulation or a qualitative shift in the immune response. Notably, protective immunity can occur independently of sustained high IFN-γ levels, as reported in *M. tuberculosis* infection (Singhal et al., 2014). The loss of AIM in *M. tuberculosis*-infected mice was associated with a moderate increase in CD4 T cell numbers and IFN-γ expression in the lung, with no significant effect on overall immune cell dynamics (Cardoso et al., 2024), highlighting the differences in host immune responses between *M. tuberculosis* and *M. avium*. However, our *in vitro* experiments showed that BMDMs increased IL-10 production upon exposure to AIM, suggesting that IL-10 produced by innate immune cells, rather than T-cell-derived IL-10, may play a critical role.

We further investigated the role of IL-10 in lipid droplet formation in AIM KO BMDMs. IL-10 secretion was decreased by AIM deletion and restored by AIM addition, demonstrating that AIM promotes IL-10 production. The finding that AIM promotes IL-10 production is consistent with that of a previous study using mouse peritoneal cells stimulated with heat-killed *Escherichia coli* (Gao et al., 2019a). In addition, IL-10-treated AIM KO BMDMs showed an accumulation of lipid droplets and an increase in CD36 expression. An increase in palmitic acid levels has been observed in the lung tissue of *M. avium*-infected mice and blood of patients with a MAC infection (Choi et al., 2024); hence, changes in lipid droplets observed in this study, at least in the *in vitro* experiment, may be CD36-dependent. Further experiments, including IL-10 inhibition, are needed to determine the extent to which AIM-induced lipid droplet formation is dependent on IL-10.

Statins are cholesterol-lowering drugs that inhibit 3-hydroxy-3-methylglutaryl coenzyme A reductase, which is involved in cholesterol synthesis in the host. Statins promote bacterial clearance in *M. tuberculosis* infection by promoting phagosome maturation and intracellular cholesterol reduction in macrophages (Parihar et al., 2014; Dutta et al., 2016, 2020; Guerra-De-Blas et al., 2019). Our study, along with previous studies (Caire-Brändli et al., 2014; Choi et al., 2024), revealed that lipid droplet formation in macrophages supports the intracellular growth of *M. avium*, suggesting that statins may enhance natural immunity against MAC infections. Similar to their use in *M. tuberculosis* infections, statins could be a promising therapeutic approach; however, further studies are needed to clarify their effects on MAC infections.

Our study had some limitations. First, the molecular pathway through which *M. avium* infection induces AIM expression remains unclear. Future studies should focus on elucidating the precise mechanisms underlying the interaction between bacteria and host. Understanding this pathway is crucial for planning more targeted experiments, such as AIM inhibition studies, and will be essential for further investigating the role of AIM in immune responses during infection, especially when combined with AIM overexpression models. Second, our *in vitro* experiments used mouse BMDMs. To enhance the clinical relevance of our findings, however, it will be crucial to validate these results using human cells.

Our study demonstrates the pivotal role of AIM in modulating macrophage polarization, lipid metabolism, and immune response during *M. avium* infection. Targeting AIM or its regulatory pathways may offer novel therapeutic strategies to enhance bacterial clearance and mitigate lung damage in MAC infections. Further research is needed to explore the potential of AIM inhibitors in clinical settings and their effect on host-pathogen interactions.

## Data Availability

The original contributions presented in the study are included in the article. Further inquiries can be directed to the corresponding author.
